# Thiophene-based porous organic networks for volatile iodine capture and effectively detection of mercury ion

**DOI:** 10.1038/s41598-018-32360-y

**Published:** 2018-09-19

**Authors:** Minghan Liu, Chan Yao, Chunbo Liu, Yanhong Xu

**Affiliations:** 1 Key Laboratory of Preparation and Applications of Environmental Friendly Materials (Jilin Normal University), Ministry of Education, Changchun, 130103 China; 2 Key Laboratory of Functional Materials Physics and Chemistry of the Ministry of Education, Jilin Normal University, Siping, 136000 China; 3 Institute of Green Chemistry & Chemical Technology, Jiangsu University, Zhenjiang, 212013 China

## Abstract

A series of conjugated microporous polymers containing thiophene-moieties (SCMP-COOH@1-3) was obtained by a homo-coupling polymerization reaction. Then the SCMP-COOH@1-3 were directly pyrolyzed without any templates to synthesize the porous carbon networks, named as SCMP-600@1, 2 and 3. SCMP-600@1-3 possess moderate BET surface area of 362–642 m^2^ g^−1^, have a permanent porous structure and plenty of sulfur and oxygen units in the skeletons as effective sorption sites, and display a high absorption performance for iodine vapour with an uptake up to 204 wt.%. In addition, SCMP-COOH@1-3 polymers can be used to effectively detect mercury ion from ethanol-water solution. Interestingly, under the same concentration of Hg^2+^ conditions, the detection ability of mercury ion of porous materials increased with the increase of the pore volumes and the specific surface.

## Introduction

Conjugated microporous polymers (CMPs) as a sub-class of porous organic polymers, which are usually synthesized by metal-catalysed cross-coupling polymerization to form 3D networks with extended π-conjugation and permanent porosity^1–4^. In the past few years, CMPs have attracted extensive attention in a variety of potential applications not only due to their finely tuned porosity and larger surface areas, but also they combine the extended π-conjugation skeleton and high thermal and chemical stability, such as gas storage and separation^5–7^, catalysis^8–10^, light harvesting^11–13^, drug delivery^14–16^, supercapacitors^17–19^, and iodine capture^20–23^, and so on. ^129^I is an important radioisotope in nuclear waste, which is particularly long-lived (half-life of 1.57 million years) and remains radioactive after a few thousand years, therefore, the exploration of adsorption for iodine capture is on the rise. CMPs have exhibited high efficient iodine capacities in reported research studies. For example, Li’s and Ben’s group also demonstrated that increase the surface areas of CMPs benefited to enhance the iodine uptakes^24^. Zhu’s group reported a series of charged porous aromatic polymers (PAF-23-25) with three different high affinity binding sites and capacity for iodine adsorption, which capture the iodine uptake value vary from 260–276 wt.%^25^. Faul’s group developed a series of amine functionalized CMPs with extremely high iodine affinity with uptake capacities as high as 336 wt.%^26^. Han’s group demonstrated that the CMP containing π electron enriched porphyrin and phthalocyanine exhibited high affinity to iodine, which displays excellent adsorption of iodine vapor up to 290 wt.%^27^. These reports imply that CMPs can be used as a novel class of materials for effective trapping of iodine.

**Figure 1:**
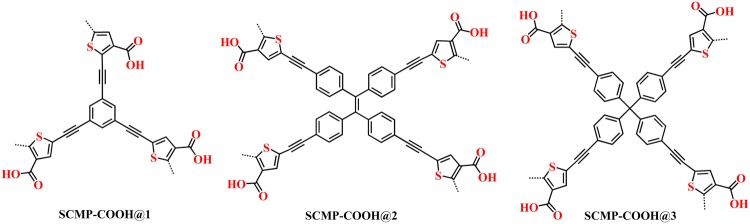
Synthetic routs of thiophene-based polymers.

Particularly, previous studies suggest that porous polymers containing such electron-rich heterocyclic have a positive impact on their iodine uptake because lone pair electrons of hetero-atoms can enhance the interaction between the adsorbents and adsorbates^20,28–31^. Therefore, introduction of some polar groups such as nitrogen or sulfur atoms into porous polymers could enhance the binding affinity between iodine and the adsorbent. For example, Li’s group synthesized a series of thiophene-beard porous organic polymer, with the highest uptake capacity up to 345 wt.%^21^. With these considerations in mind, in this paper, three thiophene-based porous materials (SCMP-600@1, SCMP-600@2, SCMP-600@3) were prepared using 2,5-dibromothiophene-3-carboxylic acid (DTCA) as a building block by the direct pyrolysis of a novel high-surface-area conjugated microporous polymers (SCMP-COOH@1-3) (Fig. 1), yielded thiophene-enriched porous carbons with incorporation of oxygen and sulfur nanoparticles. The polymers SCMP-COOH@1-3 were previously reported by our group for capture carbon dioxide (Polym. Chem. 7, 4599–4602 (2016))^32^. In this work, we have adjusted the reaction conditions of the synthesis of the polymers SCMP-COOH@1-3. Interestingly, there are great differents about the morphology of the polymers SCMP-COOH@1-3 between the two reports. The results indicated that reaction condition (solvent) plays an important role on the morphology of the polymers in the polycondensation. We have explored the detection ability of heavy metal ions for SCMP-COOH@1-3, and pore property (such as BET surface area, pore volume, pore size *et al*.) how to affect the detection ability. Furthermore, by the direct pyrolysis of high-surface-area conjugated microporous polymers SCMP-COOH@1-3, we prepared thiophene-enriched porous carbons SCMP-600@1-3 with incorporation of oxygen and sulfur nanoparticles, and the SCMP-600@1-3 display a high absorption performance for iodine vapour.

## Results and Discussion

The SCMP-COOH@1-3 polymers were prepared by taking advantage of palladium(0)-catalyzed cross-coupling polycondensation of 2,5-dibromothiophene-3-carboxylic acid (DTCA) and a number of benzene ethynyl monomers. It is different from the first report, we have utilized the 1,4-dioxane instead of DMF as a solvent. We carried out all these reactions at a fixed reaction temperature (120 °C) and reaction time (48 h).

**Figure 2:**
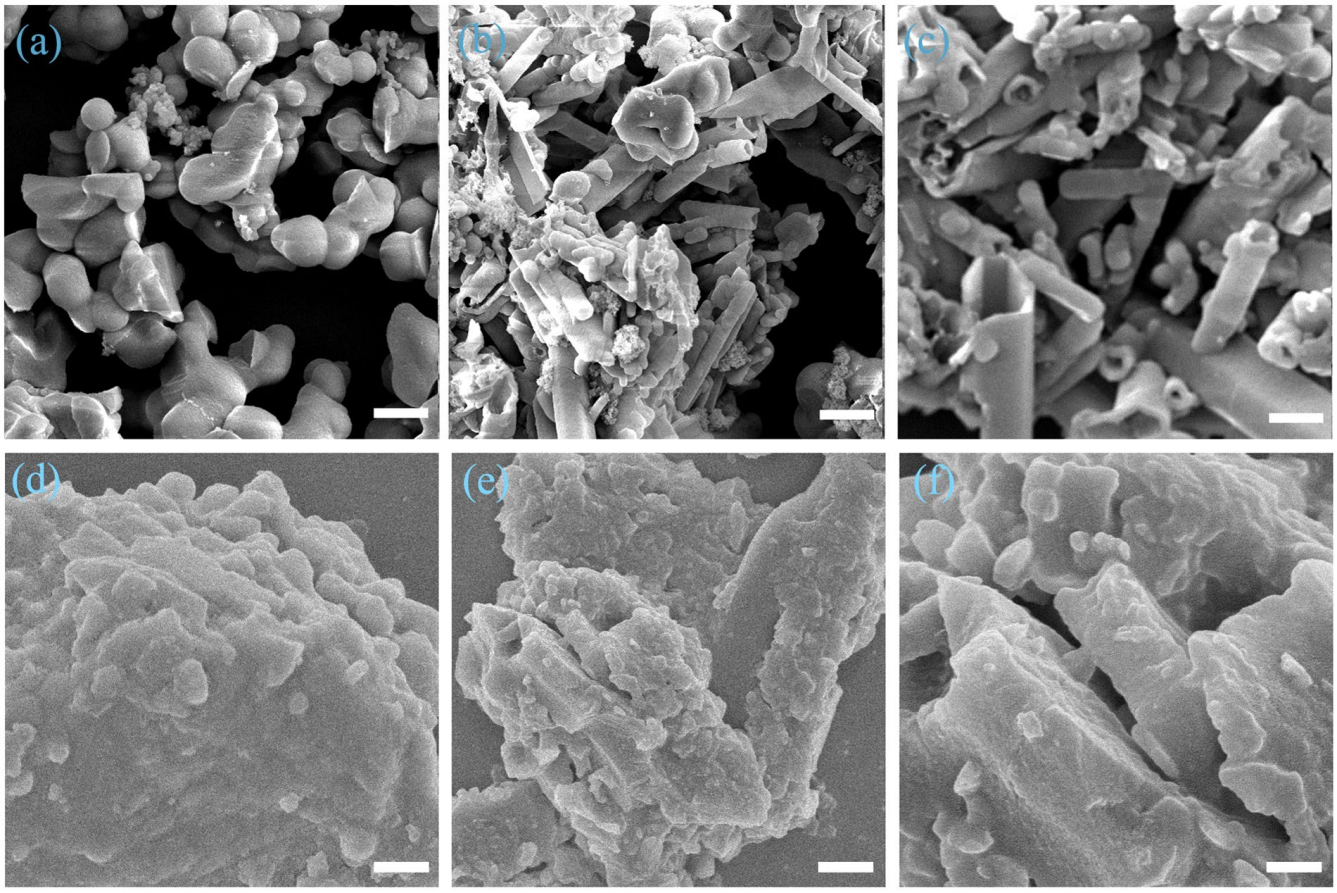
FE-SEM images of SCMP-COOH@1. (**a**) SCMP-COOH@2 (**b**) and SCMP-COOH@3 (**c**) SCMP-600@1 (**d**) SCMP-600@2 (**e**) and SCMP-600@3 (**f**) (scale bar 100 nm), respectively.

The Fourier transform infrared (FT-IR) spectrum of SCMP-COOH@1-3 revealed the presence of a characteristic O–H vibration band at 3420 cm^−1^ and a strong C=O at about 1707 cm^−1^, assignable to the carboxylic acid of thiophene moities. The vibration band of the C–Br bond at 508 cm^−1^ for the monomer is strongly diminished after polymerization (ESI, Fig. S1). This result suggests that thiophene units with carboxylic acid are covalently integrated into the porous frameworks of SCMP-COOH@1-3. The polymers showed the presence of C≡C functionalities at 2196 cm^−1^, attributable to terminal alkyne groups of acetylene benzene monomers (ESI, Fig. S1). The presence of phenylene linkages (120.3–163 ppm) and ethynylene bonds (82–100 ppm) are further confirmed by the solid-state ^13^C NMR spectrum (ESI, Fig. S2). The elemental analysis showed that the observed contents of C/H were close to the calculated value, which calculated in an ideal network with a high degree of polycondensation. It is reasonable that there are slight variations between the stoichiometry from the elemental analysis and the theoretical values^33,34^. These results demonstrated the successful fabrication of the inherently porous frameworks with a built-in carboxylic acid of thiophene unit. The amorphous nature of SCMP-COOH@1-3 was testified by powder X-ray diffraction (PXRD, ESI, Fig. S3). The thermogravimetric analysis (TGA) of SCMP-COOH@1-3 under N_2_ (ESI, Fig. S4) showed that these materials were stable to 340 °C and yield >55wt.% porous carbons when heated to 600 °C. The morphologies of SCMP-COOH@1-3 were investigated by Scanning electron microscopy (SEM). SEM of SCMP-COOH@1 displayed spherical (Fig. 2(a)), while SCMP-COOH@2 and 3 displayed tubular morphologies with several micrometers in width and up to tens of micrometers in length (Fig. 2(a–c)). Previously our group have reported the polymers SCMP-COOH@1-3 with powder morphology, which was similar to that reported for other CMP networks for capture carbon dioxide^32^. Interestingly, there are great differents about the morphology of the polymers SCMP-COOH@1-3 between the two reports. The results indicated that reaction condition (solvent) plays an important role on the morphology of the polymers in the polycondensation. SCMP-COOH@1-3 possess amorphous architecture analyzed by transmission electron microscopy (TEM) (ESI, Fig. S11(a–c)). Nitrogen sorption analysis of SCMP-COOH@1-3 gave evidence for a moderate Brunauer–Emmett–Teller(BET) surface area of about 724, 901, and 1042 m^2^ g^−1^ for SCMP-COOH@1, SCMP-COOH@2 and SCMP-COOH@3, respectively (ESI, Fig. S5). Well-defined nanopores of SCMP-COOH@1 and SCMP-COOH@2 with a size of about 1.0 nm, while the pore size of SCMP-COOH@3 centered at about 2.0 nm. The pore volumes of SCMP-COOH@1, 2, 3 are about 1.06, 1.27 and 1.48 cm^3^ g^−1^, respectively (ESI, Table S1). These results implied that different geometries of the monomer can be envisaged to tune the porous characteristics of the porous materials.

### Detection of heavy metal ions

**Figure 3:**
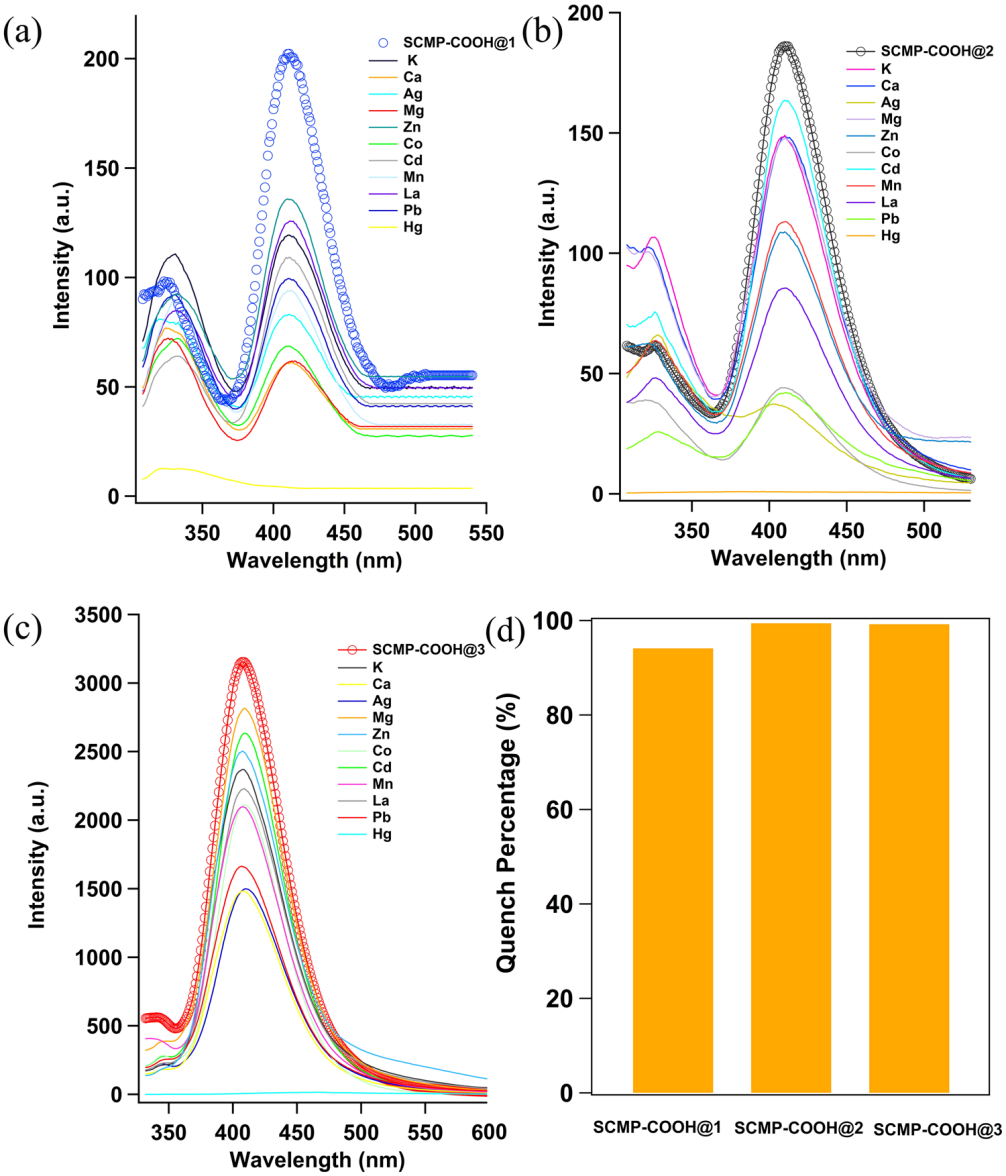
Degree of fluorescence quenching of the SCMP-COOH in ethanol-water solutions of different metal ions (10^−2^ M). (**a**) SCMP-COOH@1, (**b**) SCMP-COOH@2, and (**c**) SCMP-COOH@3. (**d**) Quench percentage of SCMP-COOH@1-3.

Water is the source of life and one of the most precious natural resources. However, with the rapid development of industry in the world, water containing heavy metal ions has become a global environmental problem because of their toxicity and biological accumulation^35–37^. Heavy metal wastewater is considered to be one of the most serious industrial wastes that are harmful to the environment and human health. Therefore, it is a quite important issue to detect and remove heavy metal ions from water before they are discharged into the environment. In this work, the SCMP-COOH@1-3 was found to be capable of efficiently detection of heavy metal ions such as Hg^2+^ ions (Fig. 3). When the SCMP-COOH@1-3 were treated in the ethanol-water (v:v = 1:9) solutions of corresponding metal salts with different concentrations, which can significantly quenched the fluorescence of the three polymers. Hg^2+^ can quench the fluorescence degree of the three CMPS up to about 99%. Compared with Hg^2+^ ions, the SCMP-COOH@1-3 exhibited low sensitivity and torpid response to other metal ions, such as K^+^, Ca^2+^, Ag^+^, Mg^2+^, Zn^2+^, Co^2+^, Cd^2+^, Mn^2+^, La^3+^, and Pb^2+^. These results clearly indicated that the SCMP-COOH@1-3 are responsive to Hg^2+^ ions (Fig. 3). The fluorescence quenching may be attributed to energy and electron transfer which was from CMPs to the metal complexes formed by the interaction between the frameworks of polymers and transition metal ions^38,39^. Besides, the metal ions may induce the aggregation of the polymer chains, giving rise to the fluorescence attenuation^38,40^. The high selectivity for Hg^2+^ is probably ascribed to several integrated factors, for instance, the suitable open pore size of the frameworks, the suitable radius, the soft Lewis acidic and oxygen-abominating character of the Hg^2+^ ion^41,42^. Especially, sulfur groups are the privileged ionophoric receptor for Hg^2+^, resulting from the distinct π-donor character, specific affinity, low-lying empty 3d orbitals of sulfur atoms^43–46^. The specific characteristics of Hg^2+^ could effectively enhance the affinity, for example, the suitable radius, the soft Lewis acidic and oxygen-abominating character. In addition, the distinct π-donor character and specific affinity of sulfur atoms could result in the better affinity of sulfur atoms for Hg^2+^. Especially, sulfur low-lying empty 3d orbitals can strongly interact with the Hg^2+^. The densely populated yet fully accessible and flexible sulfur atoms in conjunction with their remarkable affinity for Hg^2+^ are responsible for the impressive results. However, the SCMP-COOH@1-3 materials exhibited low sensitivity and torpid response to other metal ions. This is maybe these metal ions except Hg2^+^ have no or little special affinity with sulfur groups of the microporous materials. The change of fluorescence spectra maybe be caused by the interaction between the metal ions and the SCMP-COOH@1-3 microporous materials.

In this case, in order to investigate the pore property how to affect the detection ability of heavy metal of porous polymers, a series of SCMP-COOH@3 containing different surface areas and pore volumes was synthesized, named as SCMP-COOH@3-2h, SCMP-COOH@3-10h, and SCMP-COOH@3-36h, respectively. The BET surface areas of SCMP-COOH@3-2h, SCMP-COOH@3-10h, and SCMP-COOH@3-36h are 375, 603, and 914 m^2^ g^−1^, respectively (ESI, Fig. S6). The pore volumes are 0.36. 0.79, and 1.29 cm^3^/g, respectively. The pore size distribution was decreased with the reaction time prolonged. We found that the pore porosity of SCMP-COOH@3-36h is similar to that of the sample of 48 h (SCMP-COOH@3). These results implied that tuning the pore porosity of the polymers could be accomplished by using reaction of different times, which are similar to those of the previous other CMP systems. More interestingly, under the same concentration of Hg^2+^ conditions, the fluorescence quenching degree of the polymer increased with the increase of the pore volumes and the specific surface (ESI, Fig. S7). For example, when the concentration of mercury ions is 10^−8^ M, the fluorescence quenching rates of SCMP-COOH@3-2h, SCMP-COOH@3-10h, and SCMP-COOH@3-36h are 6.38%, 10.3%, and 20.4%, respectively. Increasing the concentration of mercury ions to 10^−5^ M, the fluorescence quenching rates of SCMP-COOH@3-2h, SCMP-COOH@3-10h, and SCMP-COOH@3-36h are 36.5%, 45.2%, and 58.1%, respectively. Remarkably, as the concentration of Hg^2+^ increased from 10^−5^ and 10^−2^ M, over 94% of the fluorescence for all the polymers was quenched. The high fluorescence quenching rates for Hg^2+^ in SCMP-COOH might be reasonably related to the unique high concentration of sulfur groups in the sample, which is very helpful for the coordination between the sulfurs and these heavy metal ions in the framework of the porous polymers^47,48^. The result also suggested that the detection ability of heavy metal of porous materials relating the surface areas, pore volume and pore size, as subtle variations of these parameters can lead to different properties and in turn to porous materials with different characteristics. On the other hand, in order to investigate the anti-interference ability of SCMP-COOH toward Hg^2+^, four systems containing two equal concentrations (10^−2^ M) of each metal ion (1: K^+^ and Mg^2+^, ESI, Fig. S8a; 2: Mg^2+^ and Ca^2+^, ESI, Fig. S8b; 3: K^+^ and Mn^2+^, ESI, Fig. S8c; 4: Cd^2+^ and Ca^2+^, ESI, Fig. S8d) and subsequent addition of Hg^2+^ (10^−3^ M) have been evaluated. The emission spectra of SCMP-COOH@3-36h dispersed in ethanol-water solutions containing different two kinds of metal ions and Hg^2+^ have been monitored. Interestingly, the effective fluorescence quenching could occur upon adding 10^−3^ M Hg^2+^ into the parallel tests. These results indicated that SCMP-COOH@3 possess outstanding anti-interference ability, sensitivity and selectivity in the detection of Hg^2+^ even in the complicated system.

**Figure 4:**
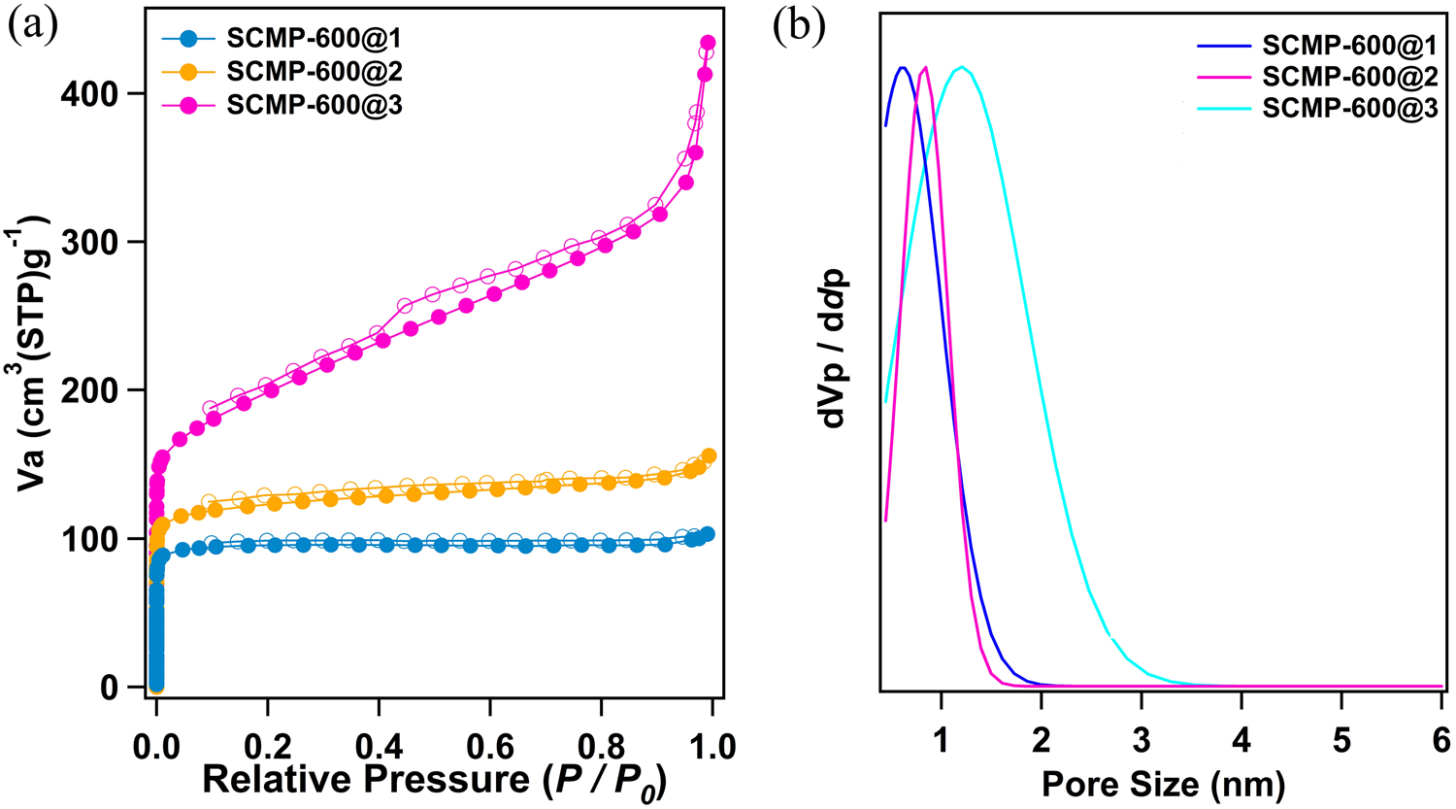
(**a**) Nitrogen sorption curves of SCMP-600@1-3 (filled circles: adsorption, open circles: desorption, STP = standard temperature pressure) and (**b**) pore size distribution of SCMP-600@1-3.

The as synthesized SCMP-COOH@1-3 networks possess a rigid conjugated backbone with a three-dimensional cross-linked architecture, such polymer precursors were subjected to template-free pyrolysis for the fabrication of heteroatom-nanoparticle-integrated sulfer enriched porous carbons. These precursors, SCMP-COOH@1-3, were pyrolysed in quartz tubes under an argon atmosphere at 600 °C for 2 h, yielded sulfer, oxygen doped porous carbon materials (SCMP-600@1-3). Simultaneously, PXRD was carried out to investigate the crystallinity of SCMP-600@1-3 samples. The wide-angle PXRD profiles of SCMP-600@1-3 exhibit the similar shapes without any sharp signals, indicating that they are amorphous random frameworks (ESI, Fig. S9). The presence of carbon, oxygen and sulfer in SCMP-600@1-3 is revealed by X-ray photoelectron spectroscopy (XPS) analysis. Both survey scan and narrow scan (S2p, O1s, C1s) were performed (ESI, Fig. S10). After pyrolysis, C1s, O1s, and S2p peaks of all samples were obviously observed in the XPS spectra (ESI, Fig. S10). The presence of oxygen and sulfer can be ascribed to thiophene unit containing carboxylic acid. scanning electron microscope (SEM) images showed irregular lumps morphologies with a length of up to tens of micrometers as well as a width ranging from hundreds of nanometers to micrometers (Fig. 2(d–f)). Transmission electron microscopy (TEM) analyses show an amorphous architeture of SCMP-600@1 3 (ESI, Fig. S11(d–f)) materials. In order to characterize the porosity parameters of pyrolysis of SCMP-600@1-3, the nitrogen sorption isotherms were measured at 77 K. Nitrogen sorption analysis (Fig. 4a) gave a specific surface area for SCMP-600@1 of 362 m^2^ g^−1^, SCMP-600@2 of 512 m^2^ g^−1^, and SCMP-600@3 of 642 m^2^ g^−1^, which are lower than those of original porous polymers SCMP-COOH@1-3 (724–1042 m^2^ g^−1^) (ESI, Table S1). The average pore size of SCMP-600@1, SCMP-600@2, and SCMP-600@3, were 0.65, 0.89, and 1.27 nm, respectively (Fig. 4b). Compare to SCMP-COOH@1-3, the pore distribution of the polymer after pyrolysis is narrower. The loss in surface area and the change in pore size distribution for pyrolysis of SCMP-COOH samples can be attributed to the structural collapse and rearrangement of SCMP-COOH frameworks during the thermal carbonization process. However, compare with SCMP-COOH@1-3, the sulfer and oxygen content of SCMP-600@1-3 decreased substantially, while the carbon content remained almost the same, at about 64% for all the samples.

### Iodine capture

**Figure 5:**
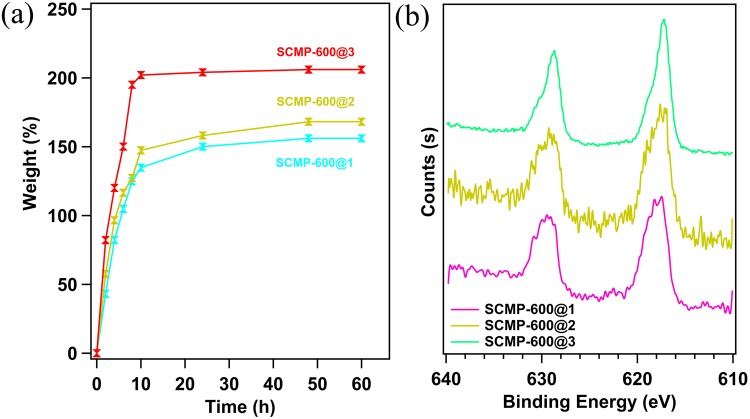
(**a**) Gravimetric iodine adsorption at a certain interval of time at 350 K. (**b**) XPS spectra of SCMP-600@1-3 after iodine capture.

More recently, as for capture and storage of volatile iodine radioiodine (^129^I and ^131^I) has been identified as one of the most dangerous species in terms of radiological effects on health and environment in case of accidental release, adsorption by using porous organic materials is considered to be an effective method. Taking advantages of excellent porous characters, remarkable high thermal stability and a large of heteroatoms in the skeleton, the resultant SCMP-600@1-3 samples should be ideal iodine absorbents which can be expected to use under harsh conditions. To our joy, we found SCMP-600@1-3 networks have highly effect on capture capacity of iodine. The samples of SCMP-600@1-3 powder (30 mg) were placed in a sealed vessel in the presence of excess iodine at 350 K and under atmospheric conditions. These conditions are typical fuel reprocessing requirements according to previous studies. The capture performance of iodine vapor was evaluated by directly weighing of the samples before and during adsorption. The adsorption capacity of iodine was calculated on the basis of the sorption curves of the samples (wt.%) plotted as a function of time (Fig. 5a). During the iodine uptake, gravity measurements were performed at different time intervals, and the results showed that the mass of SCMP-600@1-3 samples increased significantly with prolonged exposure time. From the curve, as exemplified by SCMP-600@1, there is a steep increase of iodine uptake capacity in the initial 10 h, while no significant weight gain of the sample was observed after 24 h, indicating that the system was basically saturated. Similar phenomenon were found for SCMP-600@2 and SCMP-600@3 (Fig. 5a). The saturated iodine loading is 148, 167, and 204 wt.% for SCMP-600@1, SCMP-600@2, and SCMP-600@3, respectively, which are comparable to most of the reported porous solid adsorbents for iodine capacity including zeolites, MOFs, and POPs, such as Ag-MOR (18 wt.%)^49^, ZIF-8 (120 wt.%)^50^, HCMP-3 (336 wt.%)^26^, PAF-25 (260 wt.%)^25^ and AzoPPN (290 wt.%)^27^. X-ray photoelectron spectroscopy (XPS) of the I_2_@SCMP-600@1-3 indicated that the valence of the encapsulated iodine species is zero, which confirmed that the included iodine species exists as I_2_, suggesting a physical-sorption process (Fig. 5b). Particularly, the recovered polymer was washed with ethanol to remove the adsorbed iodine, dried in vacuum, and reused in the next round of adsorption. The SCMP-600@1-3 can be efficiently recycled and reused for 5 cycles. Meanwhile, there is no significant iodine adsorption loss during the process of the cycle (ESI, Fig. S12). Previous studies have proven that the desired solid porous absorbent with high BET surface area, large pore volume and high affinity to iodine molecules would enhance the iodine uptakes of porous absorbents. It’s well-known that the capture of iodine is a trapping and physical deposition of volatile iodine into the framework of porous materials. In this case, the designed SCMP-600@3 with higher surface area and larger pore volume, which shows the highest iodine uptake as expected. These results indicated that π-π conjugated structures, high specific surface area and electron-rich building units of porous absorbents will lead to an increase in iodine capture, which is a comprehensive effect.

## Conclusions

In summary, a series of thiophene-based CMPs and porous carbon materials have been successfully developed using a building block with ligating O- and S-functionality as starting monomers. Among, SCMP-600@3 exhibited the highest iodine capture in vapour, which is comparable to those of some porous polymers, such as PAF, CMPs, and MOFs. More importantly, SCMP-600@1-3 can be recycled 5 times without obvious loss of the adsorption ability, indicating that SCMP-600@1-3 is a kind of recyclable solid absorbent. Besides, SCMP-COOH@1-3 display outstanding performances for the detection of heavy ions such as Hg^2+^. And, Hg^2+^ ions have superb anti-interference ability, sensitivity and selectivity in the detection of Hg^2+^ even in the complicated system, which is potentially important for industrial applications in water treatments. These results suggested that thiophene-based porous materials provide a good opportunity to be applied in industrial applications in the future.

## Methods

### Synthesis of SCMP-COOH@1

60 mg (0.4 mmol) of 1,3,5-triethynylbenzene and 174 mg (0.6 mmol) of 2.5-dibromothio-3-carboxylic acid are placed in a 50 mL two-necked round bottom flask and the flask is under vacuum/N_2_ Exchange 3 times under conditions. Each flask was then further individually charged with 1,4-dioxane (2 mL) and triethylamine (2 mL, Et_3_N) and the flask was further degassed by cycling multiple freeze-thaw steps. After heating to the reaction temperature, 27.7 mg (0.024 mmol) of tetrakis(triphenylphosphine)palladium(0) dissolved in 1,4-dioxane (1 mL) was dissolved in Et_3_N (1 mL). 5.7 mg (0.032 mmol) of copper(I) iodide was pumped into the flask and the reaction temperature was controlled to continue stirring at 120 °C for 48 h under nitrogen protection. The solid product was obtained by filtration, and the reaction solvent was washed successively with THF, methanol, acetone and water several times. The polymer was further subjected to Soxhlet extraction with methanol and THF, respectively, for 24 h to give SCMP-COOH@1 (91.5% yield) as a yellow solid. Elemental Analysis (%) Calculated Value. (Actual value of an infinite two-dimensional polymer) C 61.70, H 1.73. Found: C 60.58, H 2.06.

### Synthesis of SCMP-COOH@2

150 mg (0.50 mmol) of 2.5-dibromothio-3-carboxylic acid and 98.3 mg, (0.26 mmol) of 1,1,2,2-tetrakis(4-ethynylphenyl)ethane are placed in a 50 mL double-necked circle In the bottom flask, the flask was exchanged three times under vacuum/N_2_ conditions. The flasks were then re-injected into 1,4-dioxane (2 mL) and Et_3_N (2 mL), respectively, and the flask was further degassed by cycling multiple freeze-thaw steps. After heating to the reaction temperature, 17.9 mg (0.015 mmol) of tetrakis(triphenylphosphine)palladium(0) dissolved in 1,4-dioxane (1 mL) was dissolved in Et_3_N (1 mL). 3.7 mg (0.02 mmol) of copper(I) iodide was pumped into the flask and the reaction temperature was controlled to continue stirring at 120 °C. for 48 h under nitrogen protection. The solid product was filtered, and the reaction solvent was thoroughly washed with THF, methanol, acetone, and water several times in that order. The polymer was further subjected to Soxhlet extraction with methanol and THF, respectively, for 24 h to obtain SCMP-COOH@2 as a yellow-brown powder (95.8% yield). Elemental Analysis (%) C 69.81, H 2.60. Found: C 67.88, H 2.25.

### Synthesis of SCMP-COOH@3

125 mg (0.43 mmol) of 2.5-dibromothio-3-carboxylic acid and 100 mg (0.22 mmol) of tetrakis(4-ethynylphenyl)methane are placed in a 50 mL two-necked round bottom flask and the flask is under vacuum/N_2_ Exchange 3 times under conditions. Each flask was then separately charged with 1,4-dioxane (2 mL) and Et_3_N (2 mL), and the flask was further degassed by cycling multiple freeze-thaw steps. After heating to the reaction temperature, 19.9 mg (0.017 mmol) of tetrakis(triphenylphosphine)palladium(0) dissolved in 1,4-dioxane (1 mL) was dissolved in Et_3_N (1 mL). 3.1 mg (0.017 mmol) of copper(I) iodide was driven into the flask and the reaction temperature was kept under stirring at 120 °C for 48 h. The solid product was filtered, and the reaction solvent was thoroughly washed with THF, methanol, acetone, and water several times in that order. The polymer was further subjected to Soxhlet extraction with methanol and THF, respectively, for 24 h to obtain SCMP COOH@3 as a yellow powder (92.5% yield). Elemental Analysis (%) C 69.42, H 2.64. Found: C 70.66, H 2.24.

### Synthesis of SCMP-600@1-3: Template-free Pyrolysis of SCMP-COOH@1-3

Under nitrogen protection, the pyrolysis of SCMP-COOH@1-3 was performed on a quartz tube in an electric furnace. The polymer SCMP-COOH 1, 2 and 3 samples were heated from room temperature to 600 °C. at a heating rate of 3 °C/min and then pyrolyzed at 600 °C for 2 h in 400 sccm argon, respectively. The pyrolysis reaction in argon at 600 °C is denoted as SCMP-600@1, SCMP-600@2 and SCMP-600@3, respectively.

## Electronic supplementary material

Supplementary Information
