# Supplementary material for: Thiophene-based porous organic networks for volatile iodine capture and effectively detection of mercury ion
Source: Sci Rep. 2018 Sep 19;8:14071. doi: 10.1038/s41598-018-32360-y (PMC6145875; doi:10.1038/s41598-018-32360-y)
Supplement: Supplementary file 1 — Supplementary Information [file 41598_2018_32360_MOESM1_ESM.doc]

**Supporting Information**

Thiophene-based porous organic networks for volatile iodine capture and effectively detection of mercury ion

Minghan Liu, Chan Yao, Chunbo Liu and Yan-hong Xu*

Corresponding Author:

Professor Yan-Hong Xu

Collge of Chemistry, Jilin Normal University, 399 Zhuoyue Street, Changchun, 130012, China, Email: xuyh@jlnu.edu.cn

**Contents**

**Section A. Materials and methods**

**Section B. Synthetic procedures**

**Section C. FT-IR spectral profiles**

**Section D. The solid-state ^13^CCP-MAS NMR**

**Section E. PXRD patterns of SCMP-COOH@1-3**

**Section F. TGA curves**

**Section G. Pore porosity**

**Section H. Detection of heavy ion**

**Section I. PXRD patterns of SCMP-600@1-3**

**Section J. XPS spectra**

**Section K. HR-TEM images**

**Section L. Recyclability for iodine uptake**

**Section M. Supporting references**

**Section A. Materials and methods**

1,3,5-Triethynylbenzene was purchased from TCI. 2.5-Dibromothiophere-3-carboxylic acid was purchased from Alfa. Tetrakis(triphenylphosphine)palladium(0), copper(I) iodide and tetra(4-bromophenyl)methane were purchased from Aladdin. All the solvents used were purchased from Aladdin.

^1^H NMR spectra were recorded on Bruker AvanceIII models HD400NMR spectrometers, where chemical shifts (*δ* in ppm) were determined with a residual proton of the solvent as standard. Fourier transform Infrared (FT-IR) spectra were recorded on a Perkin-elmer model FT-IR-frontier infrared spectrometer. The solution UV-visible analyzer was used for shimadzu UV-3600. Solid-state ^13^C CP/MAS NMR measurements wasrecorded using a Bruker AVANCE III 400 WB spectrometer at a MAS rate of5 kHz and a CP contact time of 2 ms. Photoluminescence spectra were recorded on a shimadzu F-4600 spectrometer (JAPAN) spectrofluorometer. Field-emission scanning electron microscopy (FE-SEM) images were performed on a JEOL model JSM-6700operating at an accelerating voltage of 5.0 kV. The samples were prepared by drop-casting a THF suspension onto mica substrate and then coated with gold. High-resolution transmission electron microscopy (HR-TEM) images were obtained on a JEOL model JEM-3200 microscopy. Powder X-ray diffraction (PXRD) data were recorded on a Rigaku model RINT Ultima III diffractometer by depositing powder on glass substrate, from 2*θ* = 1.5° up to 60° with 0.02° increment. The elemental analysis was carried out on a EuroEA-3000. TGA analysis was carried out using a Q5000IR analyser (TA Instruments) with an automated vertical overhead thermobalance. Before measurement, the samples were heated at a rate of 5 °C min ^-1^ under a nitrogen atmosphere.

Nitrogen sorption isotherms were measured at 77 K with ASIQ (iQ-2) volumetric adsorption analyzer. Before measurement, the samples were degassed in vacuum at 150 °C for more than 10 h. The Brunauer-Emmett-Teller (BET) method was utilized to calculate the specific surface areas and pore volume. The nonlocal density functional theory (NLDFT) method was applied for the estimation of pore size and pore size distribution.

**Section B. Synthetic procedures**

**Synthesis of tetrakis(4-((trimethylsilyl)ethynyl)phenyl)methane^S1^**

Tetra(4-bromophenyl)methane (2.04 g, 3.16 mmol), PdCl_2_(PPh_3_)_2_ (0.135 g, 0.18 mmol), CuI

(0.024g, 0.125 mmol), and PPh_3_(0.1 g, 0.38 mmol) were put into a 250 mL round-bottom flask; then the flask exchanged 3 cycles under vacuum/N_2_, anhydrous iPr_2_NH (30 mL) and trimethylsilylacetylene (2.2 mL, 30.37mmol) was added via a syringe under the N_2_. The reaction mixture was brought to reflux at 90 °C for 24 h, and then cooled down to room temperature. Solvent was removed in vacuum, and CHCl_3_ was added to dissolve the residue and filtered through a pad of Celite. The filtrate was washed with dilute Na_2_EDTA solution and then dried over anhydrous Na_2_SO_4_; the solution was concentrated, and ethanol was added to obtain tetra(4-trimethylsilyacetylenephenyl)methane as a white solid product (80% yield). ^1^H NMR (CDCl_3_, 400 MHz): *δ*(ppm) 7.26 (d, 8H), 6.85 (d, 8H), 0.26 (s, 36H).

**Synthesis oftetrakis(4-ethynylphenyl)methane ^S1^**

NaOH (0.98 g, 24.6 mmol) was dissolved in 10 mL of CH_3_OH, then added to a solution of tetrakis(4-((trimethylsilyl)ethynyl)phenyl)methane (1.8 g, 1.2 mmol) in 20 mL of CH_2_Cl_2_, and then stirred for 6 h at room temperature. The reaction mixture was washed with water, and the aqueous phase was extracted with CH_2_Cl_2_, and the combined organic phases were washed with brine, and then dried over anhydrous Na_2_SO_4_. The solution was concentrated, and ethanol was added to the solution. Tetrakis(4-ethynylphenyl)methane was obtained as light yellow solid (83% yield). ^1^H NMR (CDCl_3_, 400 MHz): *δ*(ppm) 7.39 (d, 8H), 7.12 (d, 8H), δ 3.06 (s, 4H).

**Synthesis of *p*-Tetrabromotetraphenylethene (TBTPE)** ^S2^

Powdered 1,1,2,2-tetraphenylethene (5.00 g, 15.0 mmol) was treated with bromine (7.50 mL, 0.15 mol) and the mixture was kept for 16 h at room temperature.The resulting solid was dissolved in hot toluene (120 mL), concentrated to about 20 mL, and the precipitate was isolated. Purification using flash chromatography on SiO_2_(hexanes/CH_2_Cl_2_, 20:1 in vol.) gives TBTPE as a colorless solid (5.94 g) in 61% yield. ^1^H NMR (CDCl_3_, 400 MHz): *δ*(ppm) 7.26 (d, 8H), 6.85 (d, 8H).

**Synthesis of 1,1,2,2-tetrakis[4-(trimethylsilylethynyl)phenyl]ethene^S3^**

TBTPE (1g, 1.54 mmol) and PdCl_2_(PPh_3_)_2_ (25.2 mg, 0.036 mmol) and CuI (3.6 mg 0.0185 mmol) were put in to a 100mL round-bottom flask, then the flask exchanged 3 cycles under vacuum/N_2_, then added to 50mL diethylamine. The flask was degassed by freeze-pump-thaw for 3 times, then warmed to R.T., refilled with N_2_, trimethylsilylacetylene (1mL, 7.392 mmol) was slowly added via a syringe. The mixture was heated at 50 °C for 15 h. After this the reaction mixture was cooled to room temperature, concentrated to about 10 mL. The crude product was purified by silica gel column chromatography using hexane as eluent. A white solid was obtained (72% yield). ^1^H NMR (CDCl_3_, 400 MHz) *δ*(ppm): 7.23 (d, 8H), 6.92 (d, 8H), 0.26 (s, 36H).

**Synthesis of 1,1,2,2-tetrakis(4-ethynylphenyl)ethene^S3^**

1,1,2,2-Tetrakis[4-(trimethylsilylethynyl)phenyl]ethene (1.5 g, 2.1mmol) and THF (40 mL) were placed were put into a 250mL round-bottom flask. Then, KOH (1.8 g, 32 mmol) dissolved in 40mL of methanol was added. The mixture was stirred at room temperature overnight. After most of the solvent was evaporated, 100 mL of 1 M aqueous HCl solution was added and the mixture extracted with dichlormethane three times. The organic phases were combined and washed with water and brine and then dried over MgSO_4_.After filtration and solvent evaporation, the crude product was purified by a silica gelcolumn chromatography using hexane/dichlormethane (100:1 by volume) mixture as eluent. A yellow solid was obtained (81% yield). ^1^H NMR (400 MHz,CDCl_3_ ):*δ* (ppm) 7.28 (d, 8H), 6.93 (d, 8H), 3.06 (s, 4H).

**Synthesis of SCMP-COOH@3-2h, SCMP-COOH@3-10h, and SCMP-COOH@3-36h,.** 2.5-Dibromothiophere-3-carboxylic acid (125 mg, 0.43 mmol) and tetrakis(4-ethynylphenyl)methane (100 mg, 0.22 mmol) were put into a 50 mL two-necked round-bottom flask, then the flask exchanged 3 cycles under vacuum/N_2_. Then added to 2 mL 1,4-dioxane and 2 mL triethylamine, the flask was further degassed by freeze-pump-thaw for 3 times. When the solution had reached reaction temperature, a slurry of tetrakis(triphenylphesphine)palladium (0) (19.9 mg, 0.017mmol) in the 1 mL 1,4-dioxane and copper(I) iodide (3.1 mg, 0.017mmol) in the 1 mL Et_3_N was added, and the reaction was stirred at 120 °C under nitrogen for 2h / 10h / 36h. The solid product was collected by filtration and washed well hot reaction solvent for 4 times with THF, methanol, acetone, and water, respectively. Further purification of the polymer was carried out by Soxhlet extraction with methanol, and THF for 24 h, respectively, to give SCMP-COOH@3-2h / SCMP-COOH@3-10h / SCMP-COOH@3-36h as yellow powder. Elemental Analysis (%) C 69.11, H 3.06, O 13.90. Found: C 70.66, H 3.15, O 12.45 (SCMP-COOH@3-2h); C 69.11, H 3.06, O 13.90. Found: C 70.66, H 3.15, O 12.45 (SCMP-COOH@3-10h); C 69.11, H 3.06, O 13.90. Found: C 70.66, H 3.15, O 12.45 (SCMP-COOH@3-36h).

**Table S1 Porosity properties and gas uptake for the polymers**

| **Polymers** | *S*_BET_^a^  /m^2^ g^-1^)^a^ | *S*_micro_^b^  /m^2^ g^-1^ | V_total_^c^  /cm^3^ g^-1^ | V_micro_  /cm^3^ g^-1^ |
| --- | --- | --- | --- | --- |
| SCMP-COOH@1 | 724 | 637 | 1.06 | 0.88 |
| SCMP-COOH@2 | 901 | 833 | 1.27 | 0.92 |
| SCMP-COOH@3 | 1042 | 717 | 1.48 | 1.02 |
| SCMP-COOH@3-2h | 375 | 243 | 0.36 | 0.23 |
| SCMP-COOH@3-10h | 603 | 341 | 0.79 | 0.45 |
| SCMP-COOH@3-36h | 914 | 517 | 1.29 | 0.73 |
| SCMP-600@1 | 362 | - | 0.88 | - |
| SCMP-600@2 | 512 | - | 0.97 | - |
| SCMP-600@3 | 642 | - | 1.11 | - |

*^a^*Brunauer-Emmett-Teller surface area. *^b^*Total pore volume determined from the N_2_ isotherm at P/P_0_ =0.995. *^c^*Micro-pore volume determined from the N_2_ isotherm at P/P_0_ = 0.050.

**Section C. FT-IR spectral profiles**

**
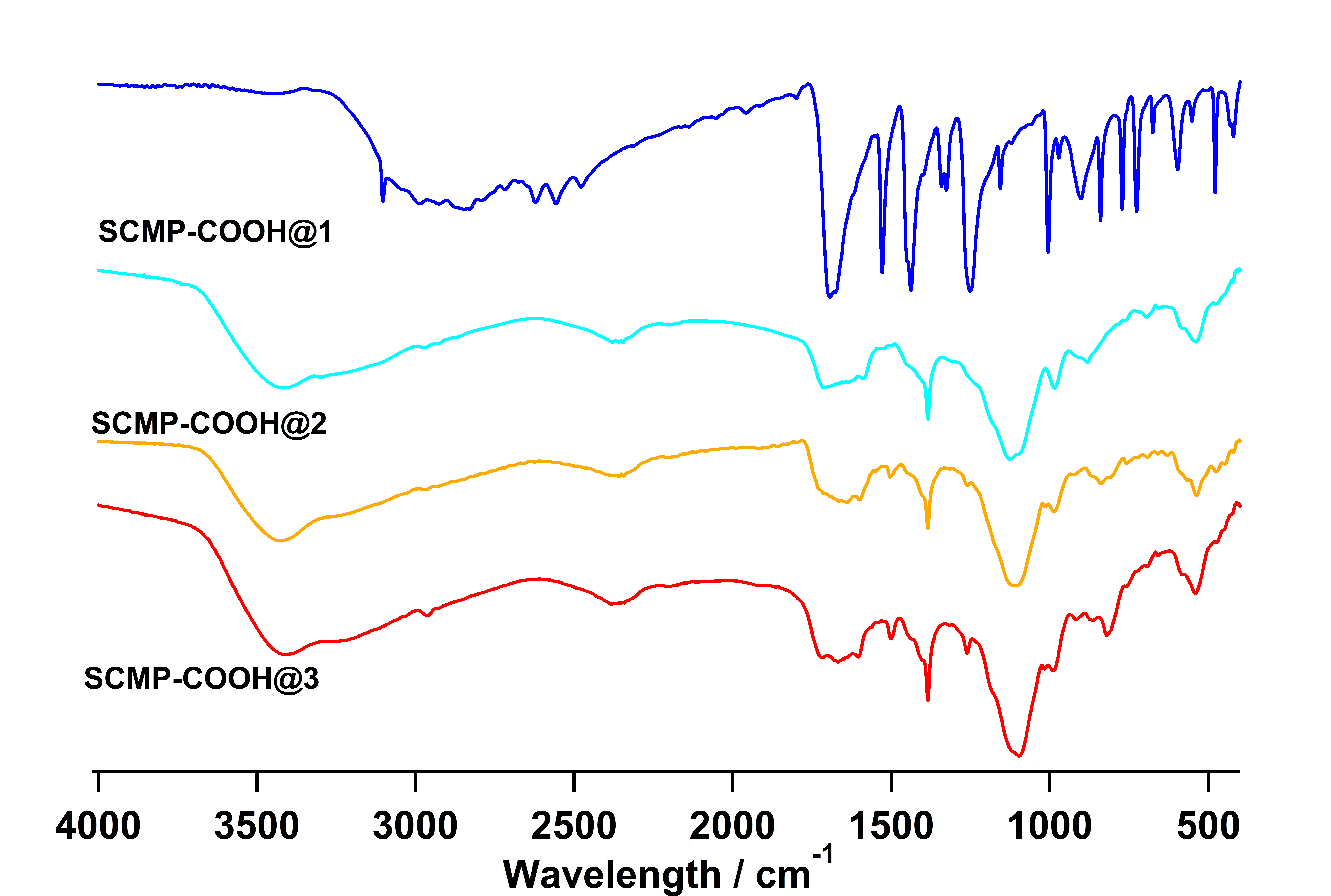
**

**Figure S1.** FT-IR spectra of 2,5-dibromothiophene-3-carboxylic acid (DTCA) (blue), and SCMP-COOH@1-3.

**Section D. The solid-state ^13^CCP-MAS NMR**


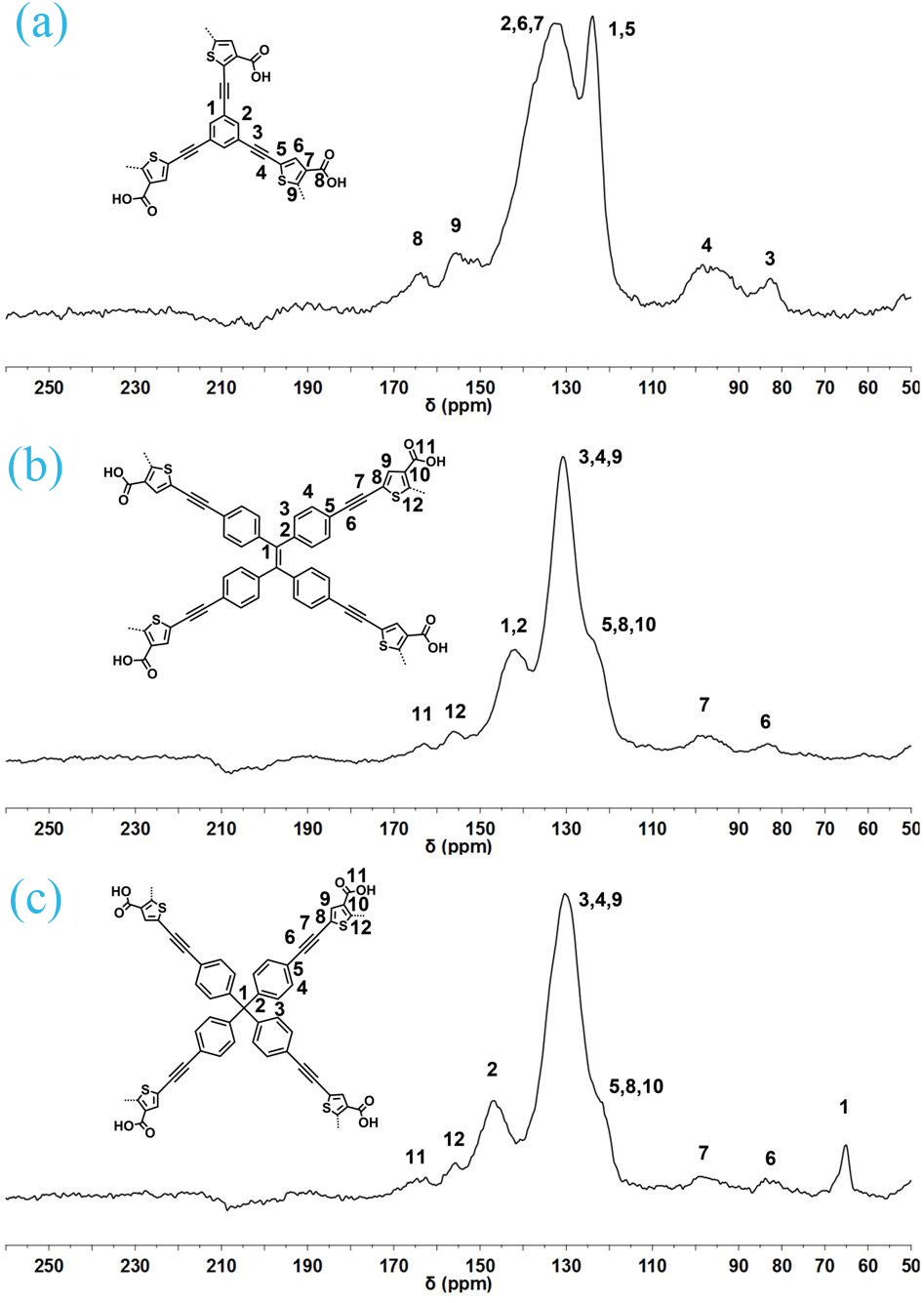


**Figure S2.** The solid-state ^13^CCP-MAS NMR of (a) SCMP-COOH@1, (b) SCMP-COOH@2, and (c) SCMP-COOH@3.

**Section E. PXRD patterns of SCMP-COOH@1-3**

**
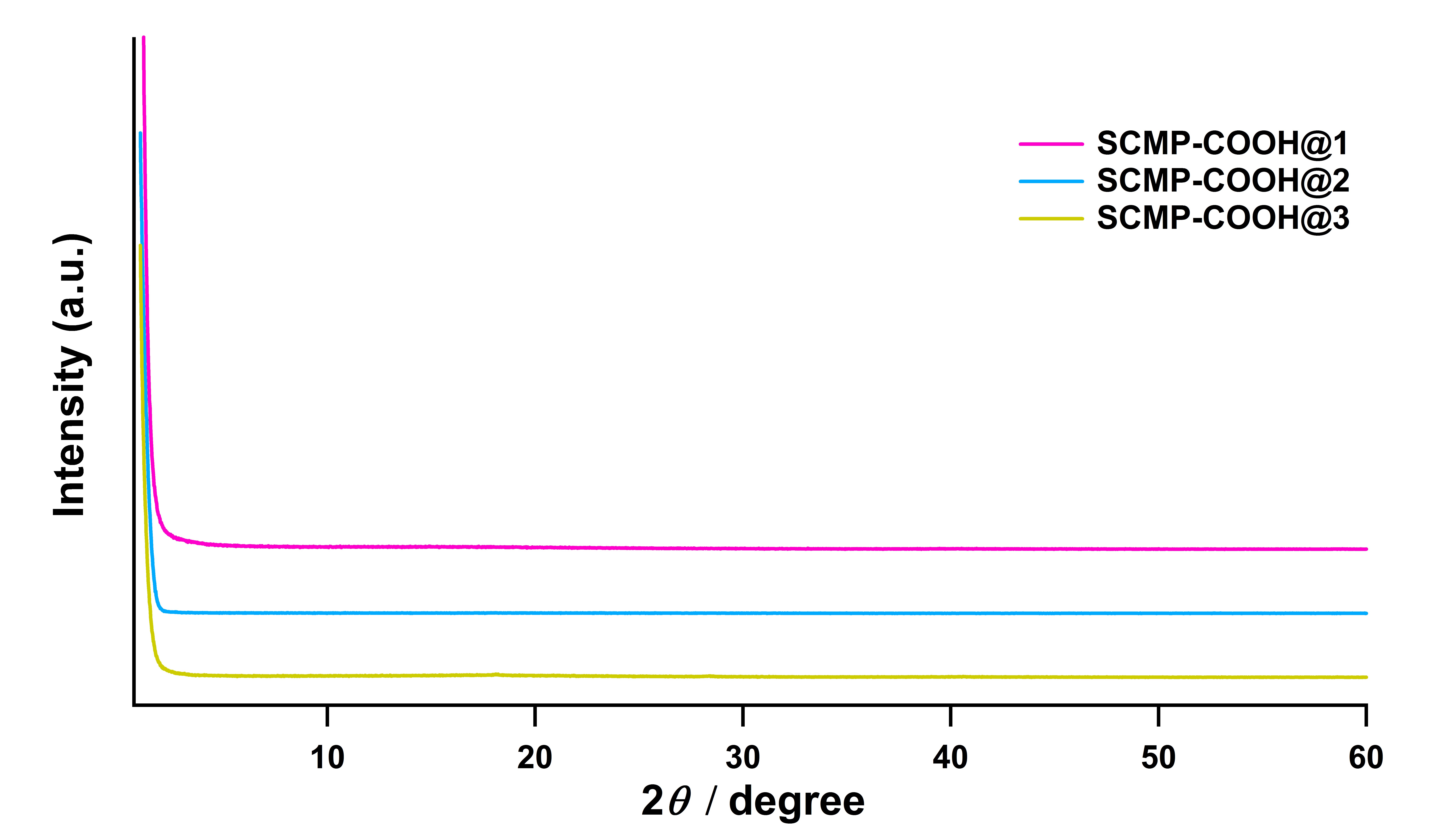
**

**Figure S3.** Powder X-ray diffraction profiles of SCMP-COOH@1-3.

**Section F. TGA curves**

**
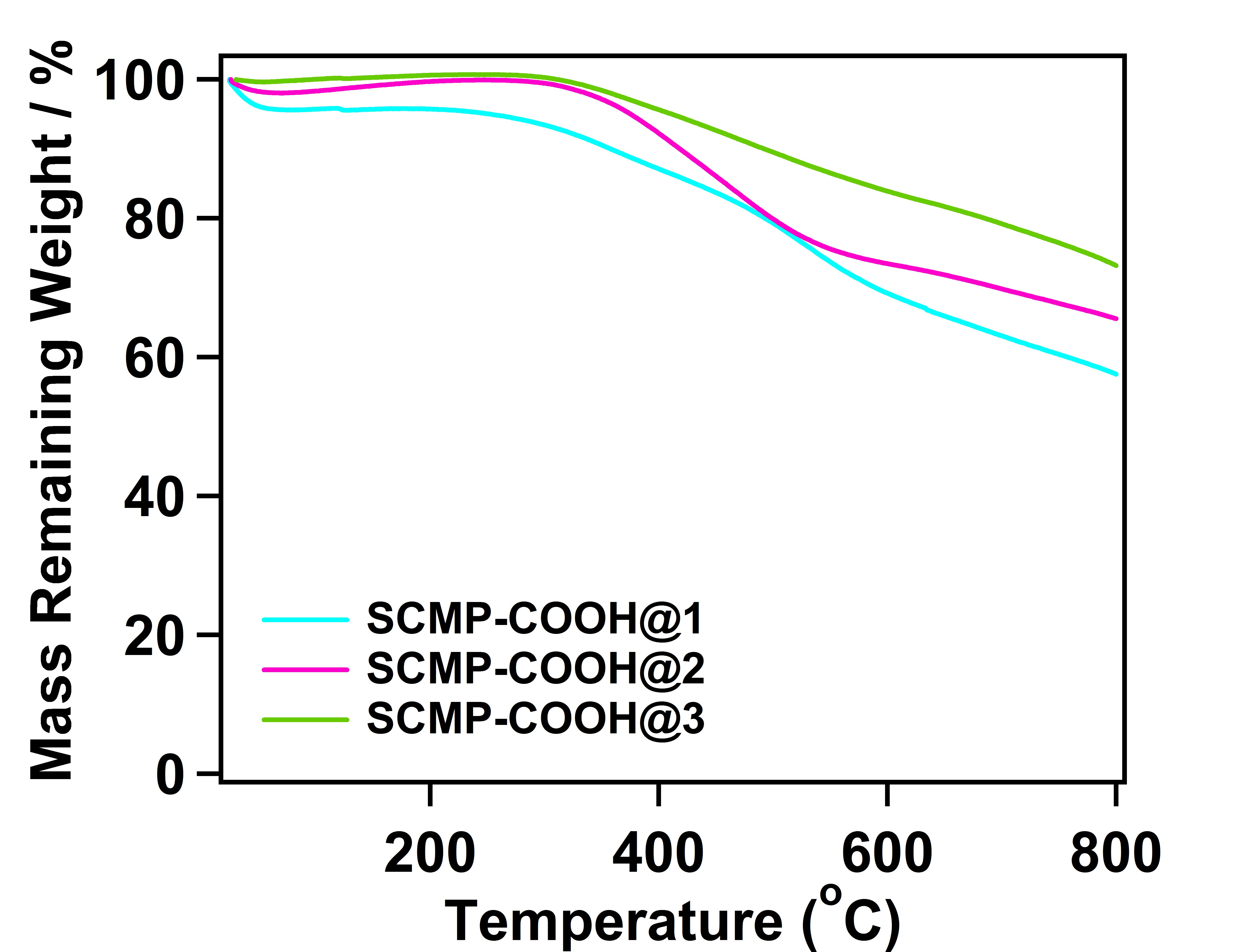
**

**Figure S4.** TGA curves of SCMP-COOH@1-3.

**Section G. Pore porosity**


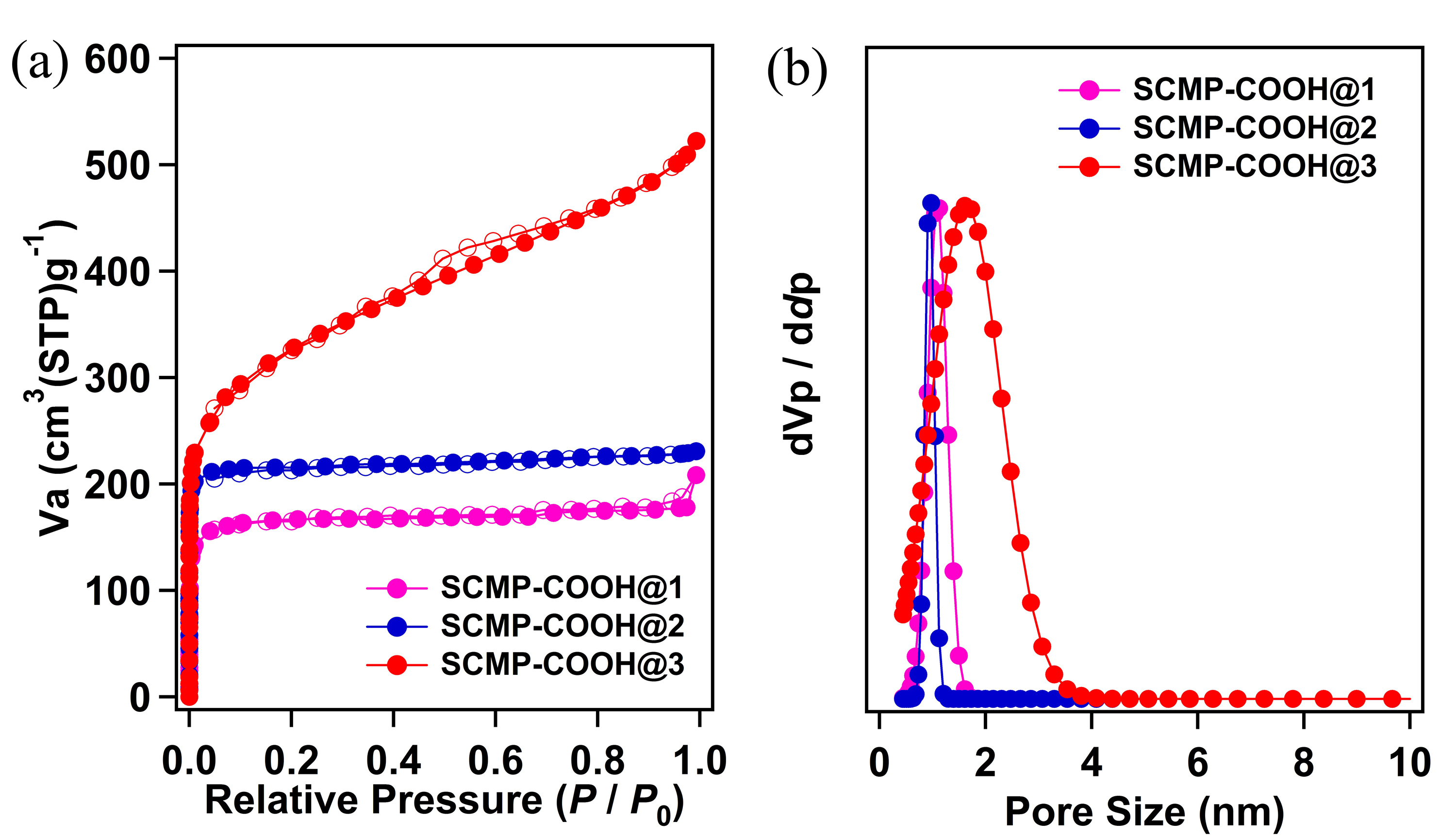


**Figure S5**. (a) Nitrogen sorption curves (filled circles: adsorption, open circles: desorption, STP = standard temperature pressure) and (b) pore size distribution of SCMP-COOH@1-3.

**Section H. Detection of heavy ion**

**
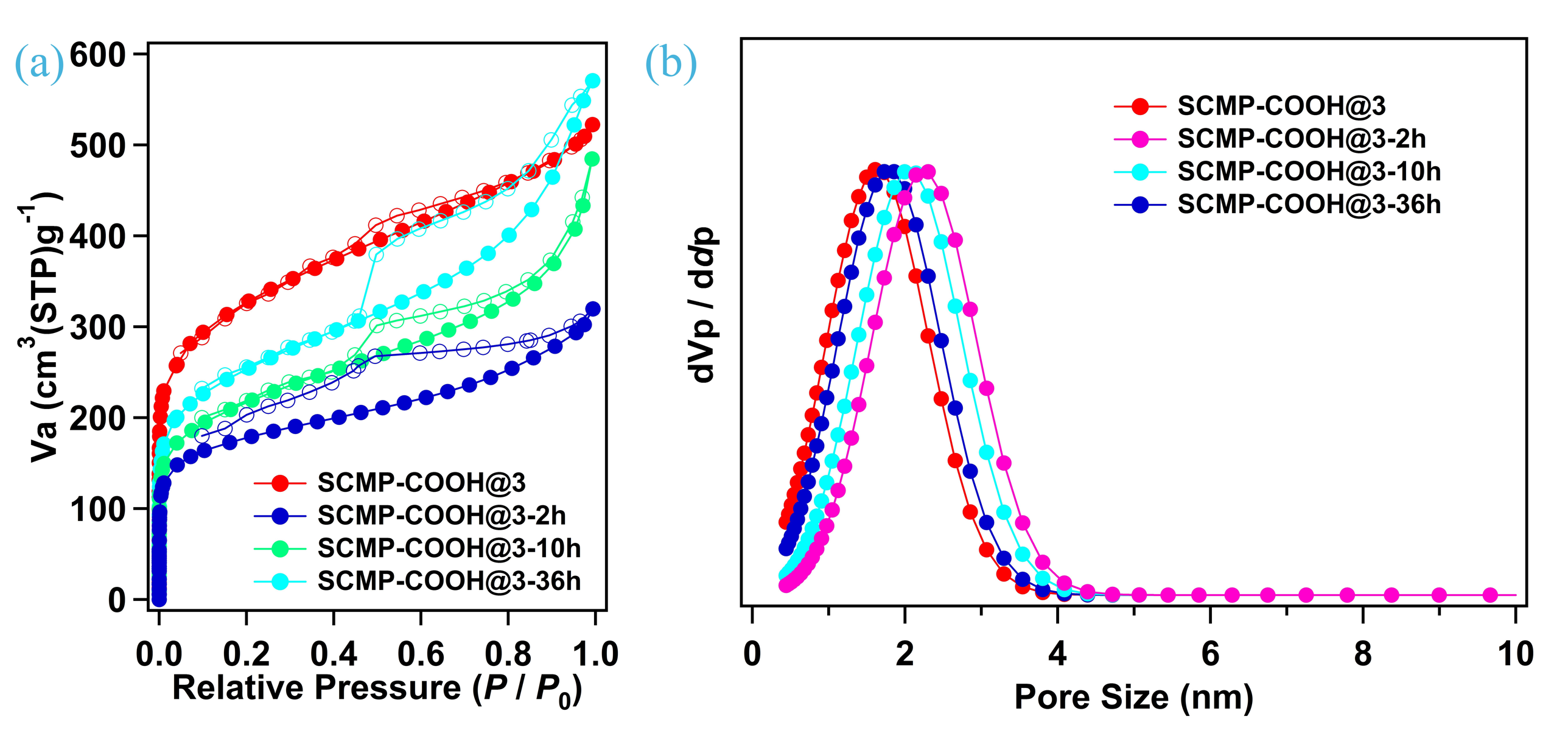
**

**Figure S6.** (a) Nitrogen sorption curves (filled circles: adsorption, open circles: desorption, STP = standard temperature pressure) and (b) pore size distribution of SCMP-COOH@3-2h, SCMP-COOH@3-10h, SCMP-COOH@3-36h, and SCMP-COOH@3.

**
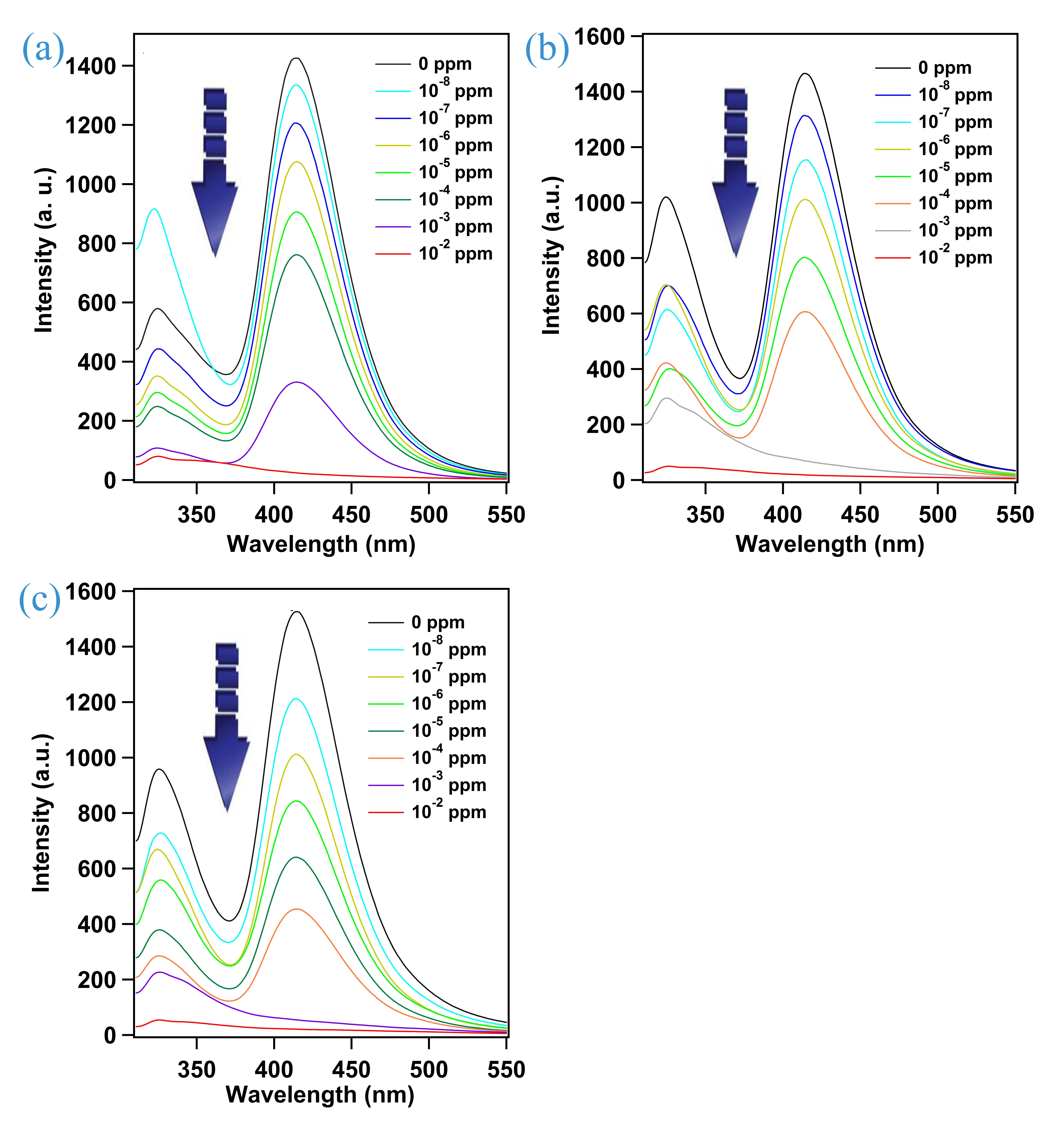
**

**Figure S7.** The fluorescence intensity of the SCMP-COOH in ethanol-water solutions with different concentrations of Hg^2+^. (a) SCMP-COOH@3-2h, (b) SCMP-COOH@3-10h, and (c) SCMP-COOH@3-36h.


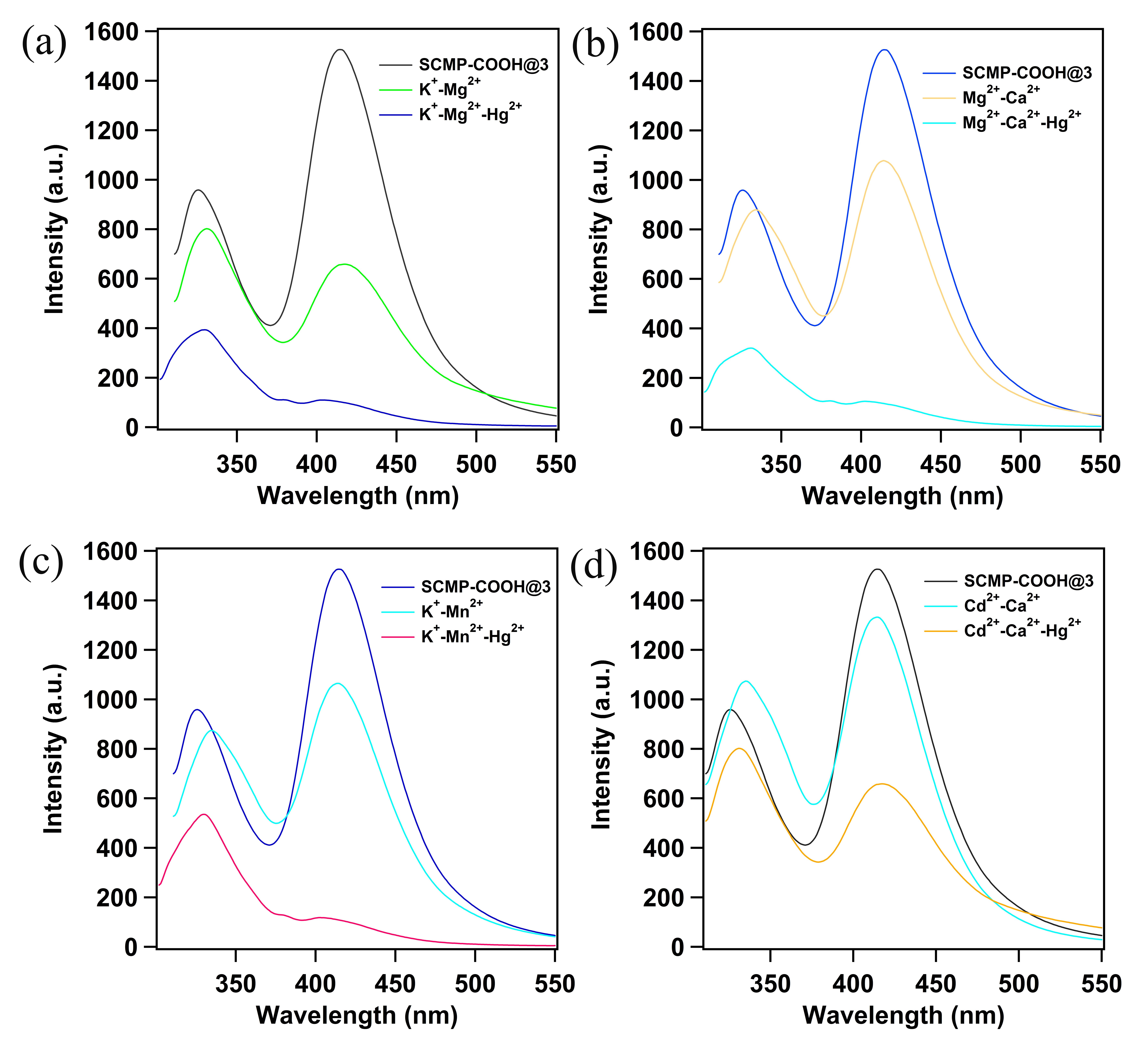


**Figure S8.** The degree of fluorescence quenching of Hg^2+^ (10^-3^ M) in ethanol-water solutions of different metal ions (10^-2^ M).

**Section I. PXRD patterns of SCMP-600@1-3**

**
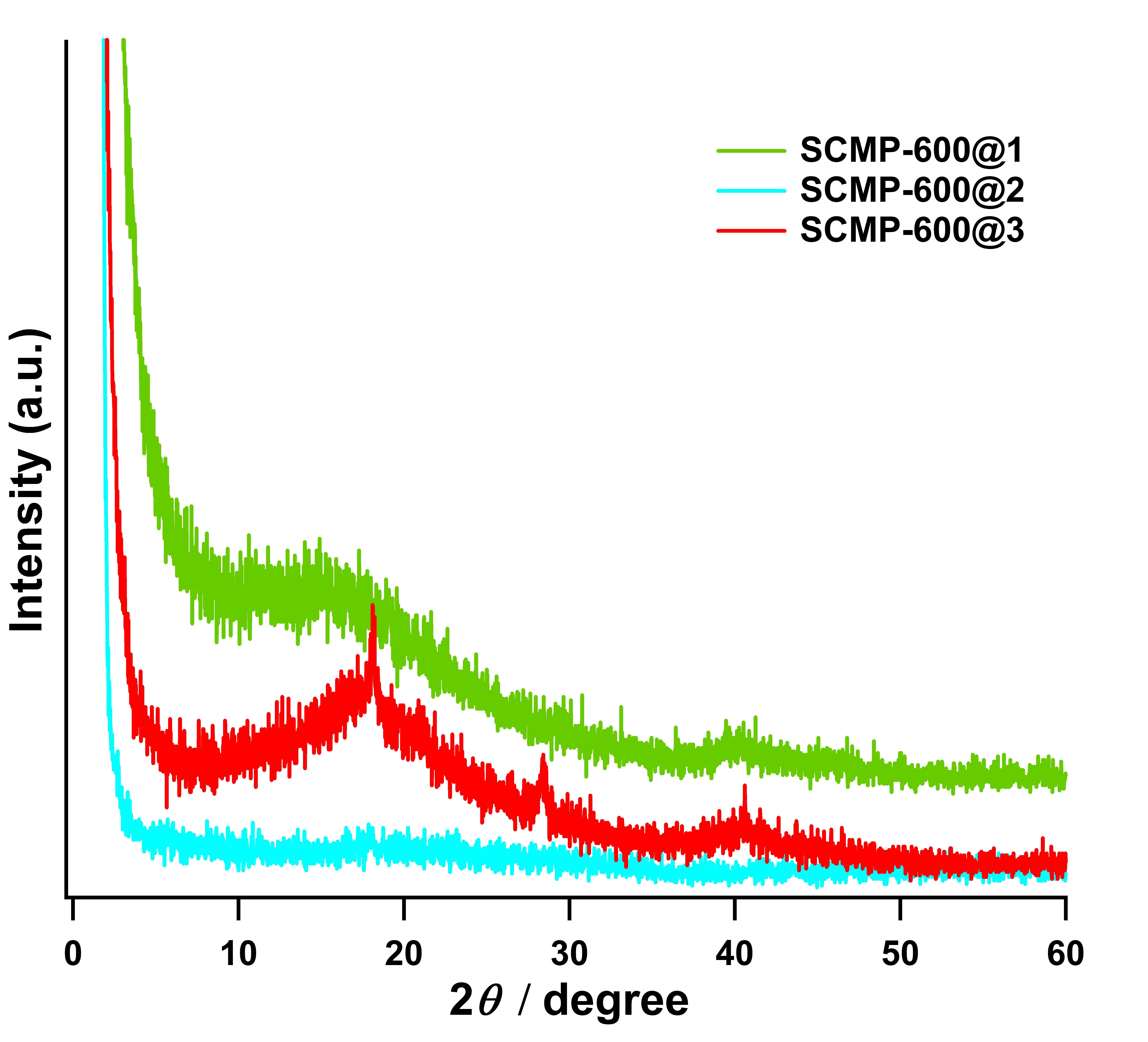
**

**Figure S9.** Powder X-ray diffraction profiles of SCMP-600@1-3.

.

**Section J. XPS spectra**


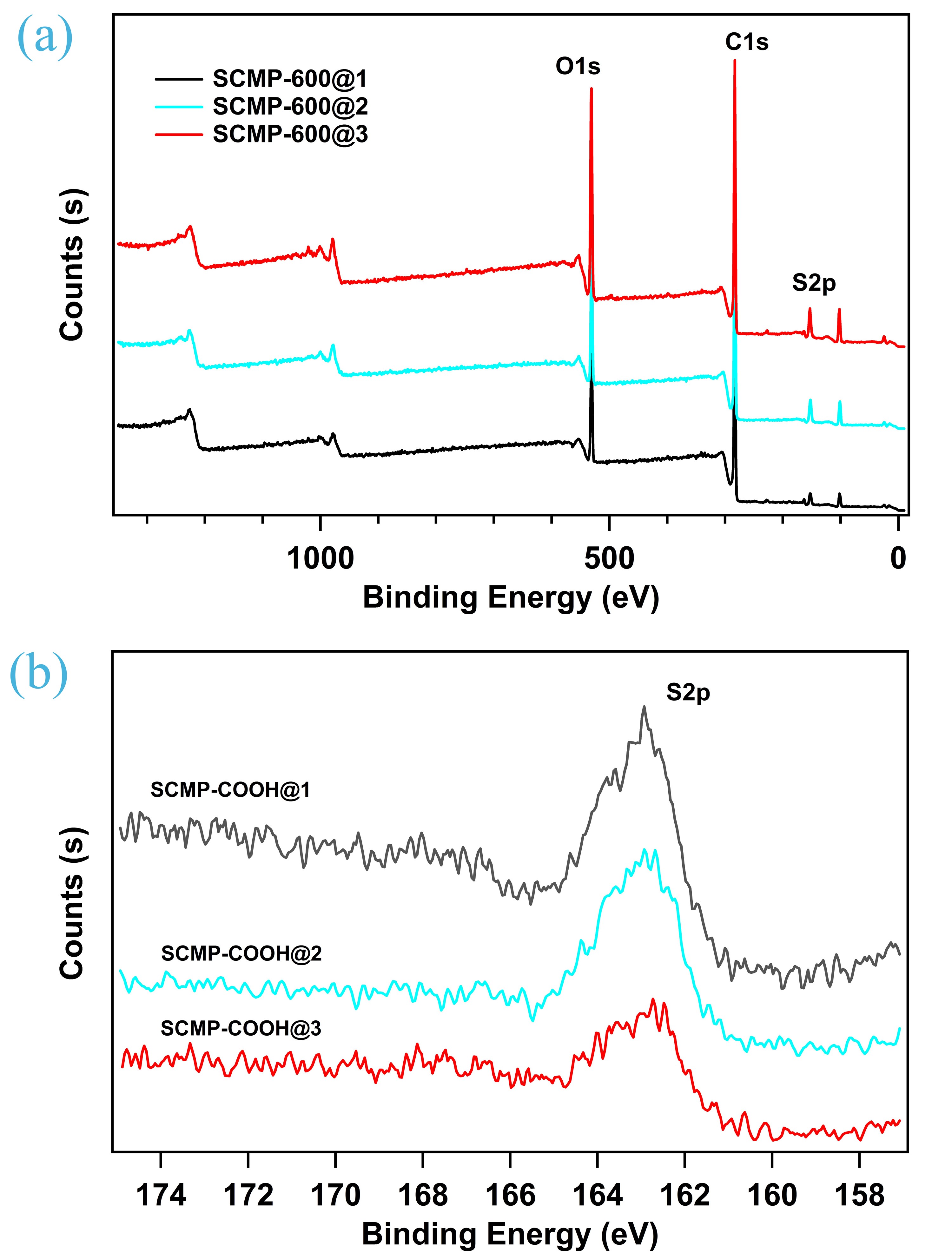


**Figure S10.** (a) XPS survey spectra of SCMP-600@1-3, (b) deconvoluted S2p spectra of SCMP-600@1-3.

**Section K.** **HR-TEM images**

**
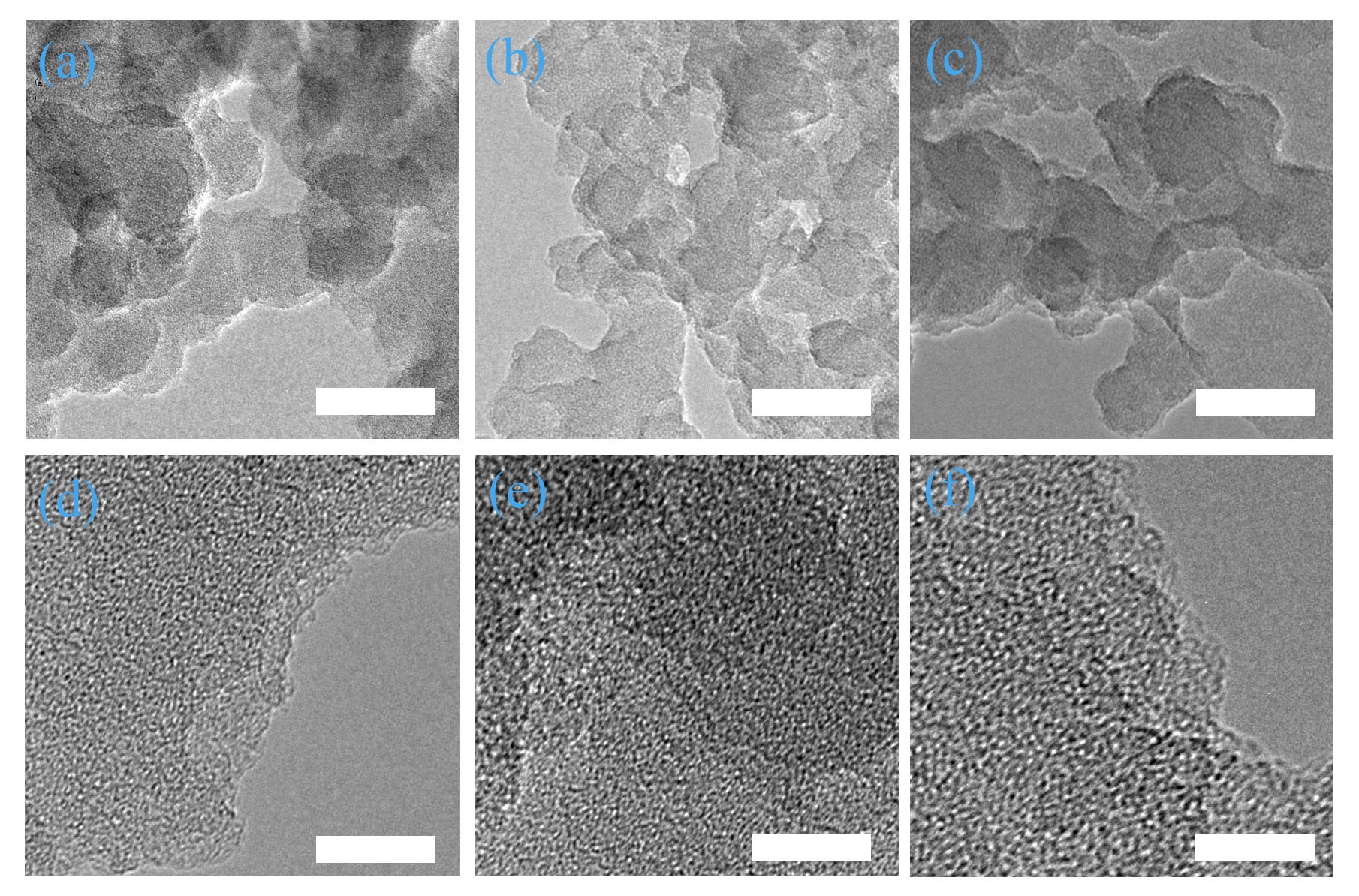
**

**Figure S11.** HR-TEM images of (a) SCMP-COOH@1, (b) SCMP-COOH@2, (c) SCMP-COOH@3, (d) SCMP-600@1, (e) SCMP-600@2, and (f) SCMP-600@3, respectively (scale bar 10 nm).

**Section L.** **Recyclability for iodine uptake**

**
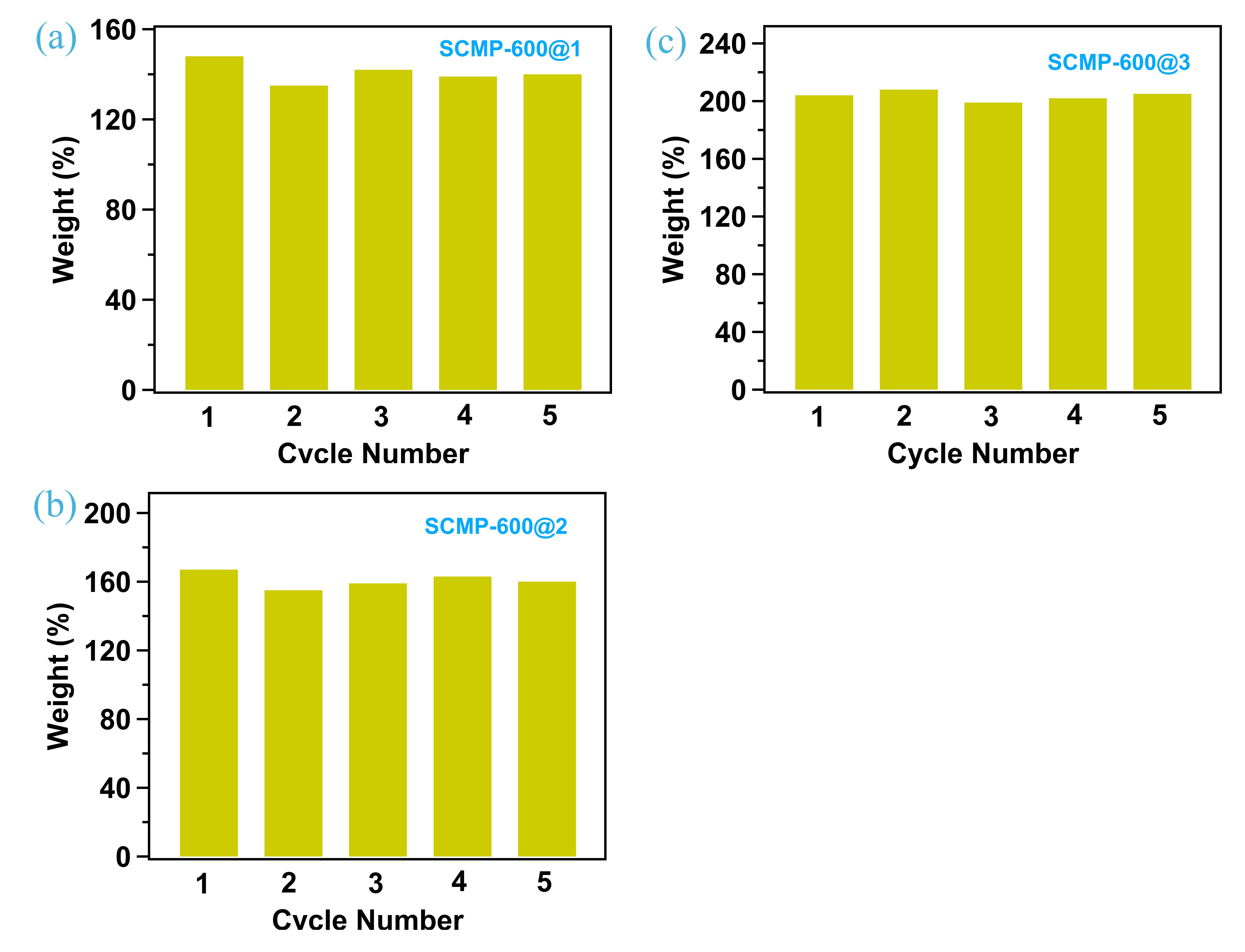
**

**Figure S12**. Reusability of SCMP-600@1-3 for iodine adsorption by vapor sublimation.

**Section M. Supporting references**

S1. Li, P. Z.; Wang, X. J.; Liu, J.; Lim, J. S.; Zou, R.; Zhao, Y. *J. Am. Chem. Soc*., **2016**, *138*, 2142-2145.

S2. Schultz, A.; Laschat, S.; Diele, S.; Nimtz, M. *Eur. J. Org. Chem*. **2003**, 2829-2839.

S3. Wang, J.; Mei, J.; Zhao, E.; Song, Z.; Qin, A.; Sun, J. Z.; Tang, B. Z. *Macromolecules*. **2012**,*45*, 7692-7703.
